# Effects of a Specific Proprioceptive Training Program on Injury Prevention and Stress in Basketball Players: A Pilot Study

**DOI:** 10.3390/jfmk10020226

**Published:** 2025-06-12

**Authors:** Vincenzo Cristian Francavilla, Giuseppe Messina, Omar Mingrino, Maria Chiara Parisi, Donatella Di Corrado

**Affiliations:** 1 Department of Medicine and Surgery, Kore University, 94100 Enna, Italy; vincenzo.francavilla@unikore.it (V.C.F.); mariachiara.parisi@unikore.it (M.C.P.); 2 Department of Human Sciences and Promotion of the Quality of Life at the San Raffaele, University of Rome, 00166 Rome, Italy; giuseppe.messina@uniroma5.it (G.M.); omarmingrino@icloud.com (O.M.); 3 Department of Sport Sciences, Kore University, Cittadella Universitaria, 94100 Enna, Italy

**Keywords:** basket, injury prevention, performance, stress levels

## Abstract

**Background**: Basketball carries a high risk of both chronic and acute musculoskeletal injuries, affecting various parts of the body. Additionally, stress is a critical factor that influences athletic performance, particularly in high-pressure sports like basketball. This study aimed to investigate the impact of a specific proprioceptive training protocol on professional basketball players. **Methods**: Thirty male basketball players (M = 21.93, SD = 3.75 years) were divided into two groups: an experimental group (n = 15) and a control group (n = 15). The experimental group completed an adapted proprioceptive training program designed to enhance position-specific skills, following their regular team training. The control group continued to follow their routine training program without any additional proprioceptive intervention. The parameters assessed included stress levels, longitudinal body axis alignment, spinal range of motion, and total plantar load distribution. These were measured at three time points: baseline (T_0_), after 4 weeks of training (T_1_), and after 8 weeks of training (T_2_). **Results**: Data analysis showed a significant reduction in stress (*p* < 0.001), postural alignment (*p* < 0.001), and spinal range of motion (*p* < 0.001) in the experimental group compared to the control group. **Conclusions**: In conclusion, the findings highlight the effectiveness of specific and detailed training programs in injury prevention, offering valuable insights for coaches and sports psychologists.

## 1. Introduction

Basketball is one of the most popular sports globally and ranks as the second most practiced team sport in Italy, with over 1.3 million participants. Characterized by dynamic physical demands, the game requires a high level of speed, agility, strength, flexibility, and balance. Players frequently engage in explosive movements such as sprinting, jumping, rapid changes in direction, and lateral shuffling—actions that substantially increase the risk of injury [1,2,3].

Success in basketball is influenced by a multifaceted combination of factors including physical fitness, technical skills, psychological resilience, and quality of coaching [4]. To perform effectively, athletes must demonstrate precise coordination, quick reactions, and the ability to adapt postural control to rapidly changing game situations [5,6]. Despite its physical and mental health benefits—such as improved body composition, cardiorespiratory health, muscular strength, and stress reduction—basketball also involves complex movement patterns that contribute to a high rate of musculoskeletal injuries [7,8,9,10].

Injuries in basketball are predominantly localized to the lower extremities, particularly the ankle and knee joints, accounting for approximately 60–70% of cases [11,12,13]. These include both acute injuries (e.g., ligament sprains) and overuse injuries (e.g., tendinopathies, stress fractures). Although prior research has explored injury patterns in basketball, there remains a lack of data linking injury risk to player roles, individual characteristics, and training variables [14]. Understanding the epidemiology of such injuries is essential for developing targeted, evidence-based prevention strategies.

As a vertical and high-intensity sport, basketball imposes substantial mechanical and physiological loads on the body. Athletes can perform up to 35–46 jumping and landing actions per game, alongside frequent lateral and rotational movements [15]. These repeated stressors may lead to muscle damage, impaired range of motion, and increased inflammatory responses, negatively affecting performance and recovery [16,17,18].

Proprioceptive training—which enhances neuromuscular control, joint stability, and dynamic balance—has proven effective in reducing injury risk in various sports settings [19,20,21,22,23]. It also contributes to improved athletic performance by fostering core strength, movement efficiency, muscular power, and rapid response capabilities [24,25]. In the context of basketball, where stability and rapid direction changes are essential, proprioceptive training offers particular value.

In addition to physical demands, basketball players face significant psychological pressures, particularly during competition. Stress, a common and often unavoidable aspect of high-level sports, can impair cognitive function, motor performance, and recovery [26,27]. When unmanaged, stress contributes not only to performance decline but also to an increased risk of injury due to its physiological effects on attention, muscle tension, and coordination [28,29,30]. Despite this, few studies have examined the role of proprioceptive training in mitigating stress responses in athletes.

Aim of the Study:

Given these considerations, the present study aimed to evaluate the effects of a targeted proprioceptive training program on both injury risk and stress levels in professional basketball players. We hypothesized that integrating the Sinergy-Mat system—a tool designed to improve balance, joint stability, and neuromuscular efficiency—would enhance postural control and reduce stress, thereby supporting overall athletic performance.

## 2. Materials and Methods

### 2.1. Participants

A group of 35 individuals was initially considered for participation in the study.

To be eligible for participation in the study, individuals had to meet the following inclusion criteria: (1) aged between 18 and 25 years of age, (2) actively involved in competitive basketball at the time of data collection, and (3) free from any known motor or neurological impairments, musculoskeletal pain, recent trauma (within the past six months), or diagnosed vestibular disorders.

Exclusion criteria included (1) a history of significant lower or upper limb injuries in the past six months, (2) current or recent participation in proprioceptive or balance-specific training programs (within the past three months), (3) any diagnosed neurological, visual, or cognitive disorders that could influence motor control or balance, and (4) the use of medications that affect neuromuscular function or balance. As a result, five participants undergoing physical therapy were removed from the study. The final sample consisted of 30 male basketball players competing at a high level. All athletes were between 18 and 25 years of age (M = 21.93, SD = 3.75) and had at least five years of experience in the sport. Each was affiliated with a sports club and followed a regular training routine averaging approximately 2.8 h per day (±0.56), four to five times per week.

Participants were randomly assigned to either an experimental group (n = 15) or a control group (n = 15), using a coin toss method. In our study, randomization was carried out as follows: for each participant, a fair coin (with two distinct sides representing the two study groups) was flipped by a researcher not involved in data collection or analysis. If the coin landed on heads, the participant was assigned to Group A; if it landed on tails, they were assigned to Group B. This procedure ensured that each participant had an equal and unbiased chance of being allocated to either group, thereby minimizing selection bias.

All research procedures adhered to the ethical standards outlined in the Declaration of Helsinki and were approved by the university’s ethics board. The study protocol received official clearance from the Departmental Research Committee (approval number: 311/2024/MEDF-02/12). Prior to the start of the experiment, informed consent was obtained individually from all participants by a member of the research team prior to their inclusion in the study. Participants were fully briefed on the purpose, procedures, potential risks, and benefits of the study. To ensure confidentiality, all personal data were anonymized and stored securely, accessible only to authorized members of the research team.

### 2.2. Procedures

All participants underwent assessments at three time points: one day before the start of the training program (baseline, T_0_), at the end of the fourth week of sessions (T_1_), and at the conclusion of the eighth week (T_2_). At each assessment, the following parameters were measured: stress levels, alignment of the longitudinal body axis (in degrees), spinal range of motion (percentage), and total plantar load distribution (percentage). All evaluations were carried out in the same laboratory under consistent conditions (room temperature maintained at 21 °C, consistent lighting and time of day), and by the same team of researchers. To reduce external variables, testing was scheduled outside of the competition period to avoid potential distractions.

The experimental group performed a specific proprioceptive training protocol (integrating the Sinergy-Mat system) three times per week for eight weeks, following their regular team training sessions. After completing the initial four weeks of sessions, participants were instructed to pause training for three days before resuming with the final four weeks of the protocol. The control group continued to follow their routine training program without any additional proprioceptive intervention. The Sinergy-Mat system is a proprioceptive training platform that combines tactile, visual, and vestibular stimulation to enhance neuromuscular control, postural stability, and joint alignment. The system incorporates unstable surfaces, target-based exercises, and balance challenges that can be progressively adapted based on the user’s ability level.

#### Proprioceptive Training Protocol

The 8-week session consisted of a basketball-specific proprioceptive training protocol developed exclusively for this study (Table 1). The training was carried out three times per week, immediately following the athletes’ regular basketball practice sessions, and was conducted on the same basketball court. The primary objectives of the training protocol were to recover joint stability, enhance muscle extensibility and strength, and promote ankle joint stabilization. A total of 24 training sessions were completed. Each session included three sets of exercises performed under both static and dynamic conditions. Static balance refers to the ability to maintain an upright posture and keep the center of gravity within the base of support, while dynamic balance involves maintaining postural control during movement, especially when the base of support shifts.

Specifically, in the central part of the training session, 5 min was dedicated to ankle mobility, including rotation and flexion–extension exercises, and another 5 min to hip mobility, focusing on rotation, adduction, and abduction exercises. During each training session, the athletes also performed 20 min of proprioceptive exercises using inclined planes, unstable surfaces, and proprioceptive platforms. The remaining time was devoted to strengthening the tonic postural system and lengthening the kinetic chains.

### 2.3. Measurements

#### 2.3.1. Psychological Stress

The level of stress was assessed using the Measurement of Psychological Stress (MPS) [31], a self-administered questionnaire consisting of 9 items. It is based on different aspects related to the perception that the individual has of its condition (cognitive–affective, physiological, behavioral). The response choices make use of a Likert-type scale whose possible answers are 1–4 (from “not at all” to “very”). The scale maintains a test–retest stability of 0.68 to 0.80 under apparently constant conditions, with a Cronbach α coefficient of approximately 0.95. The internal consistency for the present study is good (α = 0.86).

#### 2.3.2. Baropodometric Platform

Postural balance was evaluated using a Footscan baropodometric platform (Sensor Medica S.r.l., Rome, Italy), featuring dimensions of 610 × 580 mm, an active surface area of 400 × 400 mm, and a thickness of 4 mm. Participants stood barefoot on the platform in a bipedal stance, with their feet positioned approximately 20 cm apart and without any external support. They were instructed to maintain a natural, upright posture while keeping their eyes open and focused on a fixed point at eye level for a duration of 51.2 s. The software continuously tracked the position of the center of pressure at a sampling rate of 100 Hz, providing measurements of the total plantar load distribution. All data were analyzed using the platform’s integrated software.

#### 2.3.3. Photogrammetry

Photogrammetry is a widely adopted investigative technique in both medical and sports settings, used to analyze posture, movement biomechanics, and musculoskeletal disorders. By combining digital imaging with advanced processing software, it allows for highly accurate and detailed measurements of body structure and movement dynamics, making it an effective tool for both the prevention and management of musculoskeletal conditions. The method involves capturing high-resolution images from multiple angles, which are then processed using specialized software to reconstruct a three-dimensional model of the subject’s body. In this study, participants were instructed to maintain a neutral, upright posture during image acquisition. This technique is particularly valuable for detecting postural asymmetries and deviations, as it enables precise assessment of the overall alignment of the body’s longitudinal axis. Photogrammetric analysis has been validated through strong correlations with radiographic methods, demonstrating a strong concurrent validity. Test–retest reliability for photogrammetric measurements of thoracic kyphosis is excellent, with an Intraclass Correlation Coefficient of 0.97.

#### 2.3.4. Spinal Mouse

Spinal range of motion was evaluated using the SpinalMouse^®^ device (IDIAG M360^®^, Mülistrasse 18, CH-8320 Fehraltorf, Switzerland), a non-invasive, computer-assisted instrument that is manually guided along the spinous processes of the spine. Measurements were taken in three standing trunk positions: neutral, maximal flexion, and maximal extension. In the neutral position, participants stood in a relaxed, upright posture, facing forward at eye level, with feet shoulder-width apart, knees fully extended, and arms resting naturally at their sides. For the maximal flexion assessment, participants were instructed to bend forward at the waist with straight legs, attempting to touch the floor with their fingertips. For the maximal extension position, participants crossed their arms over the chest and extended the trunk backward as far as possible, avoiding cervical spine involvement. In each of these positions, the SpinalMouse^®^ was guided along the length of the spine to record sagittal alignment and mobility. No warm-up activities were performed prior to testing. The SpinalMouse^®^ has indicated a strong concurrent validity for assessing spinal alignment. Intra-rater reliability for sagittal thoracic and lumbar spine measurements is excellent, with Intraclass Correlation Coefficients ranging from 0.89 to 0.99.

### 2.4. Statistical Analysis

The results are expressed as mean ± SD. A repeated-measure 2 (group: experimental vs. control) × 3 (time: T_0_ vs. T_1_ vs. T_2_) ANOVA was conducted to assess overall changes across time points and to detect differences between groups irrespective of time and the group × time interaction, which tests whether the pattern of change over time differs between the two groups. Post hoc pairwise comparisons were performed using Bonferroni correction to explore the simple effects. An effect size was used for each analysis with the eta-squared statistic (η^2^) to evaluate the practical significance of findings. The ranges for the interpretation of the effect size based on eta-squared indicated a small effect (0.01), moderate effect (0.06) and large effect (0.14) [32]. All statistical analyses were conducted using SPSS version 26 (SPSS Inc., Chicago, IL, USA), with significance set at *p* ≤ 0.05. The results are presented as mean ± SD.

## 3. Results

A total of 30 basketball players participated in the study and were randomly assigned to either the experimental group (n = 15) or the control group (n = 15). The anthropometric characteristics and baseline outcome values of the participants are presented in Table 2. Height and body weight were measured with each athlete standing upright, barefoot, and dressed in light, comfortable clothing.

Table 3 and Table 4 present the comparisons of means and standard deviations for each variable, as well as the statistically significant differences in the assessed parameters over time.

First, there were no significant differences in baseline characteristics between the groups. Specifically, for the experimental group, post hoc comparisons revealed a statistically significant decrease in stress from T_1_ to T_2_, with a large effect size (F_(1,28)_ = 21.87, *p* < 0.001, η^2^ = 0.20), as well as a significant difference at T_2_ (*p* < 0.001).

In terms of postural alignment, the experimental group showed a significant advance, with between-group differences becoming statistically significant at both T_1_ (F(_1,28_) = 20.91, *p* < 0.001, η^2^ = 0.19) and T_2_ (F(_1,28_) = 43.21, *p* < 0.001, η^2^ = 0.25), indicating a moderate to large effect on postural alignment. Furthermore, spinal range of motion significantly increased in the experimental group over the training protocol, with the most statistically significant differences observed at T_1_ (F(_1,28_) = 17.81, *p* < 0.001, η^2^ = 0.21) and T_2_ (F(_1,28_) = 22.91, *p* < 0.001, η^2^ = 0.20), confirming the positive effect of the training protocol on spinal flexibility. Finally, in terms of total plantar load distribution, post hoc comparisons across time points revealed no significant differences.

## 4. Discussion

Basketball is an intense team sport played on a court that challenges athletes with frequent bursts of high-energy movements that can significantly strain the body, potentially disturbing its internal balance [33]. As a result, players may experience reduced performance, restricted joint mobility, and heightened inflammatory and immune system activity, often associated with an increased risk of injuries and elevated psychological stress [34]. The results supported our initial hypotheses, demonstrating significant developments in the experimental group following the 8-week training protocol. These findings align with previous research highlighting the benefits of proprioceptive training in enhancing balance, coordination, motor learning, and flexibility among basketball players [35].

For instance, Zacharakis and colleagues, examining the effects of an 8-week proprioceptive training program, reported significant developments in both dynamic and static balance among basketball players [36]. Domeika and collaborators [37] and Ondra and colleagues [38], obtained the same results after an 8-week proprioceptive training program in a group of basketball players. A comparable study demonstrated that a 12-week proprioceptive training program led to advances in passing skills among basketball players [39].

Furthermore, research suggests that proprioceptive training can enhance joint stability, reduce the risk of injury, and improve dynamic neuromuscular control in athletes, ultimately contributing to better overall performance [22,40].

In the present study, the analysis of longitudinal body axis alignment and spinal range of motion revealed significant developments over time in the experimental group, whereas no changes were observed in the control group. Consistent with our findings, Gál-Pottyondy and collaborators [2] reported similar results in basketball players. Moreover, this relationship may play a meaningful role in preventing injuries and reducing injury risk [41,42,43]. In athletic settings, core stability and strength are crucial for maintaining trunk control and contributing to overall performance enhancement. When posture is poor and muscle strength is insufficient, the body initiates compensatory mechanisms to restore balance and maintain homeostasis [44]. When an athlete’s immune system is compromised and their muscles are fatigued, they become more susceptible to illness and injury. This heightened risk is reflected in lower extremity injuries, particularly ankle sprains, which continuously impose both physiological and psychological stress on players [45,46]. Increased spinal mobility and postural alignment can enhance biomechanical efficiency, reduce compensatory movement patterns, and lower the risk of overuse injuries, particularly in high-impact sports like basketball.

In the present study, the experimental group demonstrated better emotional stability, particularly in terms of stress management and impulse control. They reported lower levels of stress compared to the control group. These results may be attributed to the specific exercises performed, including proprioceptive stimulation under controlled conditions, balance training on unstable surfaces, and other activities aimed at improving balance. Such exercises are particularly effective in improving athletic performance, especially during high-pressure game situations or when athletes must execute complex movements in challenging or unstable positions—scenarios often associated with elevated psychological stress [47]. Even modest reductions in perceived stress can have meaningful benefits for performance, recovery, and injury prevention in the demanding context of professional sports. Proprioceptive training has been shown to be especially beneficial for improving dynamic joint stability. However, our results indicate that this specific training did not lead to significant developments in load distribution or reduce the asymmetry between the left and right foot at any point during the study. The lack of significant improvement in load distribution could be attributed to some factors. First, the measurement sensitivity of the equipment may not have been sufficient to detect subtle changes. Finally, the duration and intensity of the intervention may not have been enough to induce the necessary physiological changes, which may suggest that either the intervention duration was insufficient for measurable changes in this metric or that plantar load is less sensitive to proprioceptive training compared to balance or spinal measures.

In conclusion, our results highlight the importance of incorporating proprioceptive training into regular training routines. Failure to do so can increase the risk of ankle sprains, which in turn negatively impacts performance.

### Limitations and Future Research

While the findings of this study provide valuable insights into the effectiveness of proprioceptive training for professional basketball players, several limitations should be acknowledged. First, the randomization method used—a simple coin toss—although acceptable for a pilot study, may have introduced group imbalances due to its basic nature. A more rigorous approach, such as block or stratified randomization, would be advisable in future studies to ensure better allocation and group comparability.

Second, the absence of assessor blinding is a notable limitation, particularly in relation to subjective outcome measures such as stress. Without blinding, both participants and assessors may have been influenced by expectations regarding the intervention, increasing the risk of bias and potentially affecting the objectivity of the results. Future studies should incorporate blinded assessment protocols to enhance methodological rigor. The relatively small sample size (n = 30) also limits the generalizability of the findings. Such a sample may not adequately capture the variability present in broader athletic populations. Therefore, these results should be interpreted cautiously, and future research should aim to include larger and more diverse samples to improve external validity.

Additionally, the use of a baropodometric platform, while helpful for assessing postural control, may not be sufficiently sensitive to detect subtle or incremental changes in load distribution over a short-term intervention. Longer interventions or more sensitive biomechanical assessment tools may yield more robust insights into neuromuscular adaptations. This study exclusively involved male athletes, which further limits the generalizability of the findings. Given the potential for sex-based physiological and psychological differences, future studies should include female participants to evaluate whether similar effects can be observed across genders.

The measurement of stress relied on self-report using the MPS scale, which is inherently subject to various biases, including social desirability and recall bias. Participants may have responded in ways they perceived as appropriate or favorable rather than providing entirely accurate reflections of their actual stress levels. Employing objective or multi-method approaches to assess psychological outcomes would strengthen future research in this area.

Furthermore, the study did not systematically control for or monitor external factors such as overall training load, nutrition, sleep quality, or recovery practices. These variables may have independently influenced both performance and stress-related outcomes, and their absence represents a confounding factor.

A specific limitation lies in the lack of objective monitoring of training load during the intervention period. Although both the experimental and control groups followed their usual basketball training routines, the addition of proprioceptive training in the experimental group may have led to a higher total training load. Conversely, the control group might have compensated for the lack of additional intervention through informal increases in training volume. However, training variables such as intensity, frequency, and perceived exertion were not systematically tracked, limiting our ability to isolate the effects of the proprioceptive training itself. Future research should utilize objective tools—such as training logs, heart rate monitors, or wearable tracking technologies—to quantify and compare training exposure across groups more precisely.

In summary, while our study contributes meaningful evidence regarding the potential benefits of proprioceptive training, it also highlights several methodological and contextual gaps that warrant further exploration.

Future studies should examine the long-term effects of proprioceptive training on injury prevention and athletic performance. A longitudinal design would allow researchers to determine whether the benefits observed are sustained over time or require periodic reinforcement. Moreover, it would be valuable to investigate the impact of proprioceptive training across different sports, age groups, and competitive levels (e.g., youth, amateur, or elite athletes) to assess its generalizability. Finally, expanding the study population to include female athletes in order to explore potential sex-based differences in response to proprioceptive training and utilizing wearable technology (e.g., GPS trackers, accelerometers, heart rate monitors) to objectively quantify and monitor training load, intensity, and recovery are worth consideration.

## 5. Conclusions

Our findings suggest that proprioceptive training may offer significant benefits for enhancing athletic performance and potentially reducing injury risk, particularly in dynamic sports like basketball. The broader significance of this research lies in its potential impact on training methodologies in sports science. Incorporating proprioceptive exercises into regular training routines could provide athletes with an accessible and effective tool for enhancing both performance and injury prevention. Given the high incidence of injuries in sports like basketball, this could lead to more sustainable careers and improve the overall safety of athletes. Moreover, these findings could be valuable not only for athletes but also for individuals in rehabilitation settings, where proprioceptive training could assist in recovery and the prevention of further injury.

## Figures and Tables

**Table 1:** The training protocol.

Time	Exercises
**Warm-up (10′)**	Progressive running until optimal body temperature is reached
**Mobility (10′)**	Calf mobility (2 reps ×10 each side)
	Hip mobility (2 reps ×10 each side)
**Proprioceptive Work (20′)** *using Sinergy-Mat platforms*	Squat on balance disc (bipodal)—3 reps ×10
	Pistol squat on balance disc (monopodal)—3 reps ×10—5 reps ×(L/R)
	Elastic band oscillations—frontal—3 reps ×10
	Elastic band oscillations—lateral—3 reps ×10
**Postural Work (10′)**	Standing stretch (touching toes) with wall support—3 reps ×10
*(Cool-down)*	Sitting on the floor with back against wall + forward stretch—3 reps ×10
	Spinal decompression with feet against the wall (20″)

**Table 2:** Basketball athletes’ anthropometric characteristics and baseline outcome values. Mean ± s (range).

	Experimental Group	Control Group	*p*
**Age (years)**	22.92 ± 4.64	21.73 ± 4.71	0.641
**BMI (kg/m^2^)**	24.42 ± 1.2	23.81 ± 1.1	0.582
**Weight (kg)**	87.78 ± 5.22	91.56 ± 4.82	0.673
**Height (cm)**	191 ± 7.19	195 ± 8.14	0.537
**Stress**	81.95 ± 18.38	79.87 ± 16.67	0.683
**Alignment of the** **longitudinal body axis**	1.38 ± 0.51	1.28 ± 0.58	0.586
**Spinal range of motion**	64.27 ± 9.33	61.67 ± 9.73	0.648

**Notes:** Statistically, no differences were found between these groups respect to age, height, body mass or weight.

**Table 3:** Mean (±SD) for assessed parameters across session and group.

Parameters		T_0_		T_1_		T_2_	
		EG	CG	EG	CG	EG	CG
***Stress levels***		81.95 ± 18.38	79.87 ± 16.67	75.06± 16.25	82.16± 17.93	59.61± 6.15 **	87.64± 18.53
*Alignment of the longitudinal body axis*		1.38 ± 0.51	1.28 ± 0.58	0.37± 0.45	1.38 ± 0.61	0.27 ± 0.45 **	1.35 ± 0.60
*Spinal range of motion*		64.27 ± 9.33	61.67 ± 9.73	68.33± 9.22	61.40 ± 9.43	75.60± 8.79 **	61.26 ± 9.41
*Total plantar load distribution*	Right foot	52.30 ± 5.30	52.40 ± 5.16	52.11± 4.34	52.41 ± 4.97	51.80 ± 2.55	52.53 ± 5.09
	Left foot	47.70 ± 5.29	47.60 ± 2.97	47.89 ± 4.09	47.59 ± 2.89	48.20 ± 2.35	47.47 ± 2.57

**Notes:** ** *p* < 0.001. EG = experimental group; CG = control group; T_0_ = baseline, T_1_ = after 4 weeks; T_2_ = after 8 weeks.

**Table 4:** Differences in assessed parameters over time.

Parameters	Effect	F	df	*p*	η^2^
***Stress Level***	Time	31.45	2, 27	<0.001	0.23
	Group	19.65	1, 28	0.07	0.17
	Time × Group	45.27	2, 27	<0.001	0.27
***Spinal range of motion***	Time	29.29	2, 27	<0.001	0.24
	Group	5.53	1, 28	0.02	0.17
	Time × Group	36.13	2, 27	<0.001	0.18
***Alignment of the longitudinal body axis***	Time	43.75	2, 27	<0.001	0.26
	Group	20.17	1, 28	<0.001	0.60
	Time × Group	85.75	2, 27	<0.001	0.35
***Total plantar distribution Right Load***	Time	0.045	2, 27	0.95	0.03
	Group	1.82	1, 28	0.18	0.04
	Time × Group	0.126	2, 27	0.88	0.03
***Total plantar*** ***distribution Left Load***	Time	0.042	2, 27	0.95	0.04
	Group	0.093	1, 28	0.76	0.04
	Time × Group	0.042	2, 27	0.96	0.03

## Data Availability

The data that support the findings of this study are available from the corresponding author (D.D.C.) upon reasonable request.
